# Hepatoprotective Effects of Royal Jelly Against Vincristine-Induced Hepatotoxicity in Rats: A Biochemical and Molecular Study

**DOI:** 10.3390/life15030459

**Published:** 2025-03-14

**Authors:** Rahime Erzincan, Cuneyt Caglayan, Fatih Mehmet Kandemir, Ebubekir İzol, Cihan Gür, Mustafa İleritürk

**Affiliations:** 1 Department of Bee and Bee Products, Institute of Science, Bingöl University, Bingöl 12000, Türkiye; rahime_490@hotmail.com; 2 Department of Medical Biochemistry, Faculty of Medicine, Bilecik Seyh Edebali University, Bilecik 11230, Türkiye; 3 Department of Medical Biochemistry, Faculty of Medicine, Aksaray University, Aksaray 68100, Türkiye; fmkandemir@aksaray.edu.tr; 4 Bee and Natural Products R&D and P&D Application and Research Center, Bingöl University, Bingöl 12000, Türkiye; 5 Department of Medical Laboratory Techniques, Vocational School of Health Services, Atatürk University, Erzurum 25240, Türkiye; cihan.gur@atauni.edu.tr; 6 Department of Animal Science, Horasan Vocational College, Atatürk University, Erzurum 25240, Türkiye; m.ileriturk@atauni.edu.tr

**Keywords:** vincristine, royal jelly, hepatotoxicity, oxidative stress, apoptosis, autophagy

## Abstract

Vincristine (VCR) is a chemotherapeutic agent classified as a vinca alkaloid. Royal jelly (RJ) is a significant bee product produced by worker bees, characterized by its high protein content. This study aims to investigate the protective effects of RJ against VCR-induced liver damage. VCR was intraperitoneally administered at a dose of 0.1 mg/kg body weight (b.w.) and RJ was orally administered at doses of 150 and 300 mg/kg b.w. Both treatments were applied to the rats on days 1–6 and 9–14. The composition of RJ was analyzed using LC-MS/MS, revealing the presence of 15 different phytochemical compounds with strong antioxidant properties. Serum samples obtained from the rats were analyzed for ALT, ALP, and AST levels. While these enzyme levels were significantly elevated in the VCR group, a notable reduction was observed following RJ administration. Additionally, SOD, CAT, GPx, and GSH antioxidant parameters, along with MDA levels, were evaluated in liver tissue samples. The results indicated a decrease in SOD, CAT, GPx, and GSH activities/levels and an increase in MDA levels in the VCR group. Furthermore, ELISA was used to assess JAK2, STAT3, and mTOR/PI3K/AKT signaling pathways. VCR administration led to a decrease in mTOR/PI3K/AKT levels and an increase in JAK2 and STAT3 levels. In addition, the mRNA transcription levels of inflammation (NF-κB, TNF-α, and IL-1β), endoplasmic reticulum (ER) stress (IRE-1, GRP78, PERK, and ATF-6), and autophagy markers (LC3A and LC3B) were examined. A significant increase in inflammation, ER stress, and autophagy-related markers was observed in the VCR-treated group. Lastly, the protein expression levels of Bax, Bcl-2, Caspase-3, and NF-κB were evaluated. VCR treatment increased Bax, Caspase 3, and NF-κB levels, whereas Bcl-2 levels were decreased. However, following RJ administration, all these parameters were reversed, demonstrating significant improvements. In conclusion, these findings suggest that RJ may exert a protective effect against VCR-induced liver damage.

## 1. Introduction

Cancer is one of the most commonly encountered diseases today. This disease is characterized by the uncontrolled proliferation of cells [1]. Although it has different types specific to the tissue in which it occurs, the treatment strategies for this disease are generally similar. The most frequently used of these treatment strategies is chemotherapy [2]. Chemotherapy is a treatment method in which a chemical drug is used, aiming either to completely eliminate rapidly proliferating cells or to halt their growth [3]. During treatment, the drugs used in chemotherapy affect not only the targeted tissue but also other tissues [4,5]. Vincristine (VCR) is one of these drugs and is used in the treatment of various types of cancer, particularly leukemia and lymphoma [6]. VCR is a natural alkaloid obtained from the leaves of the *Catharanthus roseus* plant and is the drug of choice against various malignancies [7]. VCR disrupts the polymerization of mitotic spindle microtubules, thereby arresting cell division in metaphase, which induces tumor cell death [8]. In addition, it has been reported that VCR can lead to tumor cell death through impaired microtubule assembly, intracellular transport of organelles and proteins, disturbed membrane stability, and cellular signaling [9]. Despite its potent antitumor activity, it is known to have cytotoxicity effects on normal cells. In vivo studies indicate that one of the main mechanisms underlying VCR-induced cytotoxicity is the induction of apoptosis via oxidative stress and inflammation [8,10,11]. The cytotoxic effects of VCR have been demonstrated on various cell types, such as hepatic, pancreatic, renal, and lymphocyte cells [11,12,13].

Royal jelly (RJ) is produced by the upper jaw and salivary glands of five- to fifteen-day-old worker bees to feed young bees, especially the queen bee. It has a gelatinous structure and a white-cream color [14]. It contains mainly water (60%), but also carbohydrates (15%), lipids (5%), protein (18%), vitamins, and minerals. Essential amino acid content is high. In addition to its widespread use in traditional medicine, it has antioxidant, anti-inflammatory, antitumoral, antibacterial, antiallergic, and antiaging effects [15,16,17]. Also, several studies have proven that RJ has hepatoprotective effects against liver damage caused by drugs, such as cisplatin, paracetamol, and diclofenac [18,19,20]. In another study, it was reported that royal jelly provided liver protection due to its antioxidant effects against hepatotoxicity caused by the combination of antituberculosis drugs, such as rifampicin and isoniazid, in rabbits [21]. In particular, RJ stands out among other bee products due to its exceptionally high protein and phenolic compound content, making it a more attractive option [22].

In the current study, firstly, the chemical content of RJ was determined quantitatively by the LC-MS/MS method. Then, the protective effects of RJ against vincristine-induced liver injury in rats were investigated.

## 2. Materials and Methods

### 2.1. Chemicals

Vincristine sulfate (1 mg/1 mL, Koçak Farma, Istanbul, Türkiye) was used in the study. Ethylenediamine tetra acetic acid (EDTA), reduced glutathione, bovine serum albumin, thiobarbituric acid, xanthine, 5,5-dithio-bis-(2-nitrobenzoic acid), perchloric acid, thiobarbituric acid (TBA), folin-ciocalteau reagent, reduced nicotinamide adenine dinucleotide phosphate (NADP), copper (II) chloride, sodium potassium tartarate, sodium hydroxide, and hydrogen peroxide (H_2_O_2_) were supplied from Sigma-Aldrich (St. Louis, MO, USA). Cumene hydroperoxide and trichloroacetic acid (TCA) were purchased from Merck (Darmstadt, Germany). RJ used in this study was obtained from local producers in Bingöl, a province in Eastern Türkiye. It was collected by local beekeepers in Bingöl during the spring of 2021 and stored at −20 °C until use.

### 2.2. Animals

Thirty-five male Wistar Albino rats, weighing 250–300 g, were used. The animals were bred at the Bingöl University Experimental Research and Application Center (BÜDAM). They were housed in a controlled environment with a constant temperature of 24–25 °C and a 12 h light–dark cycle (07:00–19:00 light; 19:00–07:00 dark). Before the experiments began, the rats were acclimatized for one week in their cages. During the experimental process, the rats were provided with standard rat feed and water ad libitum. All animal experiments were conducted at the BÜDAM center. The study was approved by the Bingöl University Animal Experiments Local Ethics Committee with the decision number 05/02, dated 8 September 2021 (Meeting No: 2021/5).

### 2.3. Experimental Design

The rats were divided into five groups, with each group consisting of seven rats, as follows:

Control Group: Rats received intraperitoneal (i.p.) injections of physiological saline on days 1–6 and 9–14.

RJ 300 Group: Rats were administered RJ at a dose of 300 mg/kg via gavage on days 1–6 and 9–14 [20].

VCR Group: Rats received i.p. injections of VCR at a dose of 0.1 mg/kg on days 1–6 and 9–14 [23].

VCR + RJ 150 Group: Rats received i.p. injections of VCR at a dose of 0.1 mg/kg on days 1–6 and 9–14. Thirty minutes prior to each VCR administration, RJ was administered at a dose of 150 mg/kg via gavage.

VCR + RJ 300 Group: Rats received i.p. injections of VCR at a dose of 0.1 mg/kg on days 1–6 and 9–14. Thirty minutes prior to each VCR administration, RJ was administered at a dose of 300 mg/kg via gavage.

### 2.4. Sample Collection

At the end of the study period (24 h after the final administration of VCR and RJ), the rats were decapitated under mild sevoflurane anesthesia, and liver tissues were collected. Blood samples were collected and centrifuged at 3500 rpm for 10 min to collect serum. The serum was kept at −80 °C until evaluation of liver function tests. The harvested tissues were stored at −20 °C until molecular analyses were performed.

### 2.5. Determination of Chemical Content of RJ by LC-MS/MS

**Figure 1:**
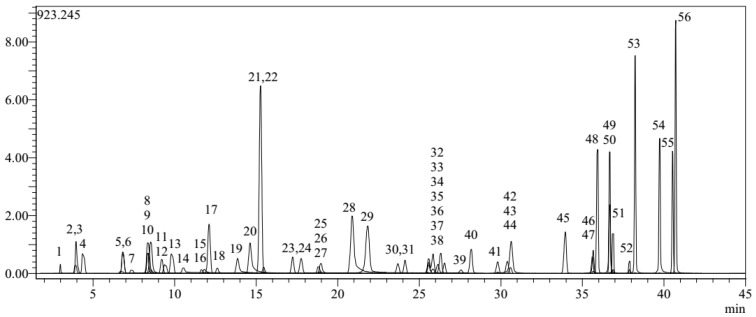
Chromatogram of standard components used in LC-MS/MS analysis.

Quantitative determination of the chemical content (flavonoids and phenolic acids) of RJ by liquid chromatography–mass spectrometry/mass spectrometry (LC-MS/MS) was performed by investigating the compounds that exhibit many biological activities in its content. In this context, quantitative analysis of 53 phytochemical compounds, including internal standards, was performed. These compounds are as follows: quinic acid, fumaric acid, aconitic acid, gallic acid, epigallocatechin, protocatechin acid, catechin, gentisic acid, chlorogenic acid, protocatechin aldehyde, tannic acid, epigallocatechin gallate, 1,5-dicaffeoylquinic acid, 4-OH benzoic acid, epicatechin, vanillic acid, caffeic acid, syringic acid, vanillin, syringic aldehyde, daidzin, epicatechin gallate, piceid, p-coumaric acid, ferulic acid, sinapic acid, coumarin, salicylic acid, cynaroside, miquelianin, rutin, isoquercitrin, hesperidin, o-coumaric acid, genistin, rosmarinic acid, ellagic acid, cosmosin, quercitrin, astragalin, nicotiflorin, fisetin, daidzein, quercetin, naringenin, hesperetin, luteolin, genistein, kaempferol, apigenin, amentoflavone, chrysin, and acacetin [24]. For LC-MS/MS analysis, RJ was first extracted and converted into a form suitable for the instrument. For this, 0.15 g of the sample was weighed and 1.5 mL of pure water was added. Then, it was vortexed for 2 min. Then, 500 µL of the extract was diluted to 5 mL with mobile phase (ammonium acetate 30 mM, pH 5 and MeOH, 50:50, *v*/*v*). Finally, it was filtered through a 0.20 µm nylon filter (to eliminate particles in the samples) and given to the device after being taken into a vial. The analysis was performed using the previously validated and established method [24]. As a result, a quantitative analysis of the compounds showing biological activity in RJ was performed and the amount of compounds was calculated. The chromatogram of the standard is also shown in Figure 1.

### 2.6. Liver Function Tests

In the autoanalyzer (Randox, Parramatta, NSW, Australia), the levels of serum aspartate aminotransferase (AST), alanine aminotransferase (ALT), and alkaline phosphate (ALP) were examined. The results for these enzymes were displayed as U/L.

### 2.7. Oxidative Stress Markers in the Liver Tissue

Before conducting the experiments, the liver tissues were frozen using liquid nitrogen and ground to a particle size of approximately 5 microns using the TissueLyser II (Qiagen) device. Ground liver tissues were weighed as 100 mg, diluted with appropriate buffers and homogenized according to specific assays to obtain a 1:10 (*w*/*v*) homogenate. For malondialdehyde (MDA), catalase (CAT), and superoxide dismutase (SOD) measurements, the homogenates were centrifuged at 3500 rpm for 15 min at +4 °C. To assess glutathione peroxidase (GPx) activity and glutathione (GSH) levels, the homogenates were centrifuged at 10.000 rpm for 20 min at +4 °C, and the resulting supernatants were used for further analysis. MDA levels, representing lipid peroxidation, were measured colorimetrically using the method of Placer, et al. [25]. The activities of SOD, CAT, and GPx were determined following the protocols of Sun, et al. [26], Aebi [27], and Lawrence and Burk [28], respectively. GSH levels were determined according to the procedure of Sedlak and Lindsay [29]. Total protein content in liver homogenates was measured using the Lowry, et al. [30] method.

### 2.8. Enzyme-Linked Immunosorbent Assay (ELISA)

All rat ELISA kits used in the study were obtained from Sunred Biological Technology (Shanghai, China) Company. All analyses were performed according to the manufacturer’s instructions. The study measured the levels of janus kinase-2 (JAK2), signal transducer and activator of transcription 3 (STAT3), mammalian target of rapamycin (mTOR), protein kinase B (AKT), and phosphoinositide 3-kinase (PI3K).

### 2.9. Total RNA Isolation and cDNA Synthesis from Liver Tissue

Total RNAs were isolated from liver tissues of rats belonging to all groups with hybrisol (HibriGen) reagent. All procedures in the RNA isolation stage were performed in accordance with the manufacturer’s instructions. After the RNA isolation steps were completed, the concentration of each sample was measured in the NanoDrop (BIO-TEK INSTRUMENTS EPOCH, Winooski, VT, USA) device. A commercial kit (iScript™ cDNA Synthesis Kit, BIO-RAD, Hercules, CA, USA) was then used to obtain double-stranded cDNA from total RNAs. According to the kit procedure, a 20 µL mixture was prepared with total RNAs, 5x iScript Reaction Mix, iScript Reverse Transcriptase, and Nuclease-free water. Afterwards, the cDNA synthesis process was completed by keeping the tubes at 25 °C for 5 min, at 46 °C for 20 min, and at 95 °C for 1 min, which are the temperatures and times specified in the procedure. These procedures were performed on the ROTOR-GENE Q (Qiagen, Hilden, Germany) device.

### 2.10. Determination of Relative mRNA Transcript Levels (RT-PCR)

In order to determine the mRNA transcript levels of the genes whose sequences are given in Table 1, the mixture was prepared by adding iTaq Universal SYBR^®^ Green Supermix (2x), forward primer (primer of the relevant gene), reverse primer (primer of the relevant gene), and RNase-DNase-free water onto the cDNA. The mixture was then subjected to temperature cycles in the ROTOR-GENE Q (Qiagen, Germany) instrument according to the manufacturer’s instructions. At the end of the process, the fold change for each gene was calculated using the 2^−ΔΔCT^ method [31].

### 2.11. Western Blot Analysis for Apoptosis

The powdered liver tissues were dissolved in 1 mL of RIPA lysis buffer (sc-24948, Santa Cruz Biotechnology, Inc., Dallas, TX, USA) that included sodium orthovanadate, phosphatase inhibitor, and protease inhibitor cocktail, and then homogenized on ice. Homogenates were centrifuged at 16,000× *g* for 20 min, and the supernatant was used for Western blot analysis. Protein concentration in homogenates was determined with the Pierce™ BCA Protein Assay Kit (Rockford, IL, USA) using bovine serum albumin (BSA) as a standard. A total of 30 µg of protein was taken from the supernatant and dissolved in Laemmli Sample Buffer, and proteins were separated in 10% sodium dodecyl sulfate polyacrylamide gel electrophoresis (SDS-PAGE). Proteins were transferred to polyvinylidene fluoride (PVDF) membranes. The membranes were then blocked in 5% BSA dissolved in tris-buffered saline (TBS-T) containing 0.1% Tween 20 for 1.5 h. After blocking, the membranes were washed 5 times for 5 min with TBS-T and specific antibodies were added and incubated overnight at +4 °C. After incubation, the membranes were washed 5 times for 5 min with TBS-T, and then goat antimouse IgG secondary antibody (Santa Cruz Biotechnology, sc-2005, 1:1000 dilution) was added and incubated for 1.5 h. After incubation with the secondary antibody, they were washed 5 times for 5 min with TBS-T. Protein bands were made visible by adding Clarity™ Western ECL Substrate (Bio-Rad, Hercules, CA, USA) chemiluminescent reagent and visualized on the ChemiDoc XRS+ (Bio-Rad Laboratories Inc., CA, USA) device. The relative density of protein bands was calculated using the ImageLab (Version 6.0.1, Bio-Rad Laboratories Inc., CA, USA) program.

### 2.12. Statistical Analysis

SPSS 20.0 package program was used for statistical analysis of biochemical data. Statistical analysis was performed using GraphPad Prism 5.01 software. Statistical differences and significance levels were determined with the “One-way Analysis of Variance (ANOVA)” test, while Tukey test was used to determine differences between groups. A *p*-value of <0.05 was considered statistically significant, and all data were presented as mean ± standard deviations (SDs).

## 3. Results

### 3.1. LC-MS/MS Analysis Results of RJ

**Figure 2:**
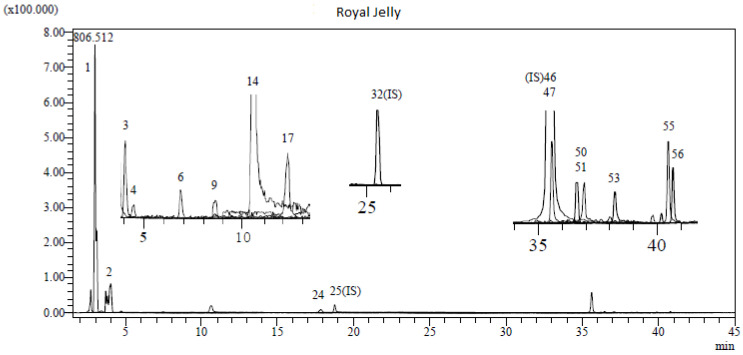
LC-MS/MS chromatogram of RJ.

Royal jelly has a high bioactive content consisting mainly of phenolic acids, flavonoids, polyphenols, fatty acids (trans-10-hydroxy-2-decenoic acid), and some vitamins (A, E and C). The chromatogram of the LC-MS/MS results of RJ is given in Figure 2. In this study, a total of 53 types of phenolic compounds were investigated in the analysis of RJ obtained from the Bingöl region in Eastern Türkiye. In the results of the comprehensive chemical content of RJ, the highest concentrations were quinic acid (6.573), fumaric acid (3.927), 4-OH benzoic acid (2.91), p-coumaric acid (0.332), aconitic acid (0.043), protocatechuic acid, (0.043), quercetin (0.037), caffeic acid (0.023), chlorogenic acid (0.019), gallic acid (0.017), chrysin (0.012), acacetin (0.011), luteolin (0.005), genistein (0.004), apigenin (0.003) (mg analytes/g sample), respectively. The results of the LC-MS/MS analysis of RJ are given in Table 2. In this study, 15 different phytochemicals were detected in RJ. Therefore, it is thought that the rich antioxidant content of RJ is due to these compounds.

### 3.2. Effects of RJ on VCR-Induced Liver Function Parameters

**Figure 3:**
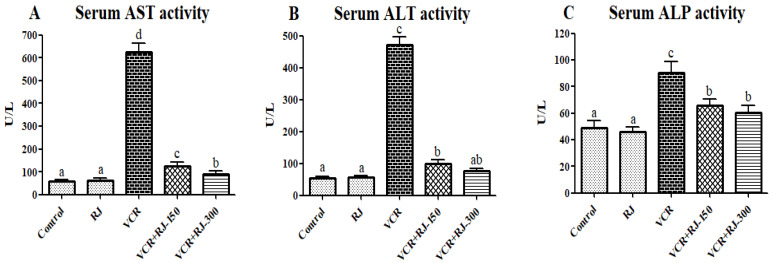
(**A**) Effect of RJ on VCR-induced serum AST level in rats. (**B**) Effect of RJ on VCR-induced serum ALT level in rats. (**C**) Effect of RJ on VCR-induced serum AST level in rats. It was found that serum levels of AST, ALT, and ALP were significantly increased in the VCR-treated group compared to the control group. However, treatment of RJ (150 and 300 mg/kg) significantly decreased the levels of AST, ALT, and ALP as compared to the VCR-treated group (*p*  <  0.05). All data were expressed as mean ± SD. Different letters (a–d) on the columns show a statistical difference (*p* < 0.05).

The effects of RJ treatment and VCR injection on hepatic injury markers, such as ALT, AST, and ALP, were presented in Figure 3A–C. VCR application alone increased serum ALT, AST, and ALP concentrations compared to the control and RJ groups. However, treatment with RJ at two doses 150 and 300 mg/kg significantly (*p* < 0.05) reduced the elevated levels of the above-mentioned serum liver enzymes.

### 3.3. Effects of RJ on VCR-Induced Oxidative Stress and Lipid Peroxidation in Liver Tissue

**Figure 4:**
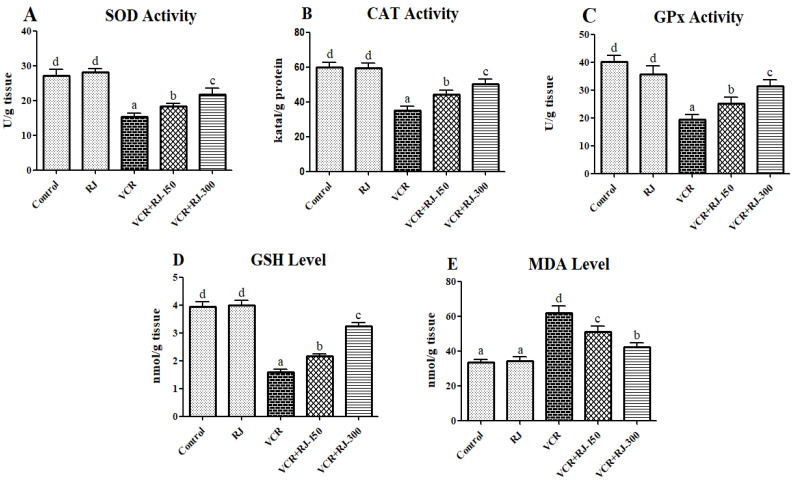
(**A**) Effect of RJ on VCR-induced liver SOD activity in rats. (**B**) Effect of RJ on VCR-induced liver CAT activity in rats. (**C**) Effect of RJ on VCR-induced liver GPx activity in rats. (**D**) Effect of RJ on VCR-induced liver GSH level in rats. (**E**) Effect of RJ on VCR-induced liver MDA level in rats. The activities of SOD, CAT, and GPx and the level of GSH were found to be decreased and the MDA level was found to be significantly increased in the VCR-treated group compared with the control group (*p* < 0.05). On the other hand, treatment with RJ (150 and 300 mg/kg) significantly decreased the level of MDA and also increased the levels of GSH and activities of SOD, CAT, and GPx as compared to the VCR group (*p*  <  0.05). All data were expressed as mean ± SD. Different letters (a–d) on the columns show a statistical difference (*p* < 0.05).

The injection of VCR was found to significantly elevate MDA levels, indicating enhanced lipid peroxidation in liver tissue, while also reducing the activities of antioxidant enzymes (SOD, CAT, and GPx) alongside GSH levels. In contrast, RJ administration notably decreased lipid peroxidation, as evidenced by reduced MDA levels. Additionally, RJ treatment significantly enhanced the activities of SOD, CAT, and GPx, as well as increased GSH levels compared to the VCR group (*p* < 0.05). A comprehensive summary of lipid peroxidation and antioxidant marker levels in liver tissue is provided in Figure 4A–E.

### 3.4. Effects of RJ and VCR on Relative mRNA Transcript Levels of NF-κB, TNF-α, and IL-1β Genes in Liver Tissue

**Figure 5:**
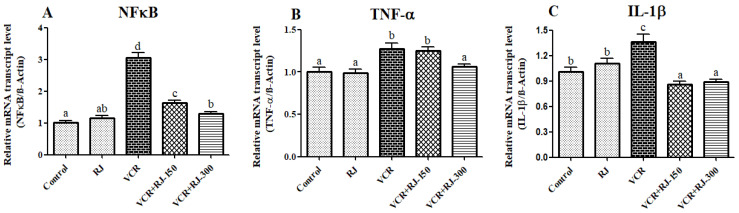
Effects of RJ and VCR treatments on NF-κB, TNF-α, and IL-1β mRNA transcription levels in liver tissue. (**A**) NF-κB mRNA transcript levels, (**B**) TNF-α mRNA transcript levels, and (**C**) IL-1β mRNA transcript levels. It was found that there were significantly increased NF-κB, TNF-α, and IL-1β mRNA transcript levels in the VCR-treated group as compared with the control group (*p* <  0.05). However, treatment of RJ (150 and 300 mg/kg) significantly decreased the mRNA transcript levels of NF-κB, TNF-α, and IL-1β compared to the VCR-treated group (*p* <  0.05). Values are expressed as mean ±  SD. Different letters (a–d) on the columns show a statistical difference (*p*  <  0.05).

According to the data presented in Figure 5A–C, it was observed that NF-κB, TNF-α, and IL-1β were upregulated in the liver tissues of rats undergoing VCR (*p* < 0.05). These markers were significantly suppressed after the animals were treated with RJ. When the doses of RJ were compared, it was seen that the 300 mg/kg dose was more effective on NF-κB (*p* < 0.05) and TNF-α (*p* < 0.05), while the mRNA transcript levels of IL-1β did not make a significant difference between the doses. It is also among the data obtained that 150 mg/kg dose did not affect TNF-α compared to the VCR group.

### 3.5. Effects of RJ on VCR-Induced PI3K/Akt/mTOR Pathway in Liver Tissue

**Figure 6:**
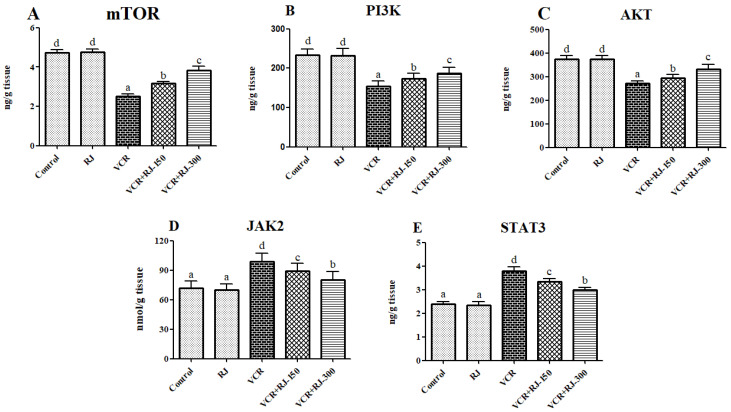
(**A**) Effect of RJ on mTOR levels in VCR-induced liver injury in rats. (**B**) Effect of RJ on PI3K levels in VCR-induced liver injury in rats. (**C**) Effect of RJ on AKT levels in VCR-induced liver injury in rats. (**D**) Effect of RJ on JAK2 levels in VCR-induced liver injury in rats. (**E**) Effect of RJ on STAT3 levels in VCR-induced liver injury in rats. The levels of PI3K, AKT, and mTOR were found to be decreased and the levels of JAK2 and STAT3 were found to be significantly increased in the VCR-treated group compared with the control group (*p* < 0.05). Co-treatment with RJ (150 and 300 mg/kg) significantly increased the level of PI3K, AKT, and mTOR and also decreased the levels of JAK2 and STAT3 compared to the VCR group (*p*  <  0.05). Values are expressed as mean ± SD. Different letters (a–d) indicate statistical difference among the groups (*p* < 0.05).

In the liver tissues, mTOR, PI3K, and AKT levels were analyzed following VCR and RJ treatments (Figure 6A–C). Compared to the control group, the VCR-only group exhibited a significant decrease in mTOR, PI3K, and AKT levels (*p* < 0.05). The RJ-only group did not cause a significant change compared to the control group; however, when compared to the VCR group, a significant increase was observed (*p* < 0.05). Additionally, in the VCR + RJ-150 and VCR + RJ-300 groups, mTOR, PI3K, and AKT levels were significantly higher compared to the VCR group.

### 3.6. Effects of RJ on VCR-Induced JAK2 and STAT3 Levels in Liver Tissue

Compared to the control group, the VCR-only group exhibited a significant increase in both JAK2 and STAT3 levels (*p* < 0.05). The RJ-only group did not cause a significant change compared to the control group; however, when compared to the VCR group, a significant reduction was observed (*p* < 0.05). Additionally, in the VCR + RJ-150 and VCR + RJ-300 groups, both JAK2 and STAT3 levels were significantly lower compared to the VCR group (Figure 6D,E).

### 3.7. Effects of RJ and VCR on Relative mRNA Transcript Levels of ATF-6, PERK, IRE1, and GRP-78 Genes in Liver Tissue

**Figure 7:**
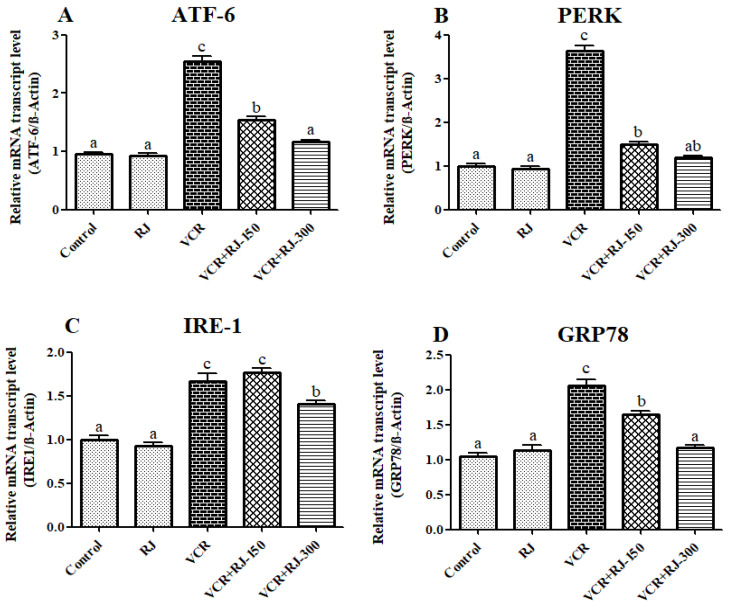
Effects of RJ and VCR treatments on ATF-6, PERK, IRE-1, and GRP78 mRNA transcription levels in liver tissue. (**A**) ATF-6 mRNA transcript levels, (**B**) PERK mRNA transcript levels, (**C**) IRE1 mRNA transcript levels, and (**D**) GRP78 mRNA transcript levels. Values are expressed as mean ±  SD. Different letters (a–c) on the columns show a statistical difference (*p*  <  0.05).

Relative mRNA transcript levels of ATF-6, PERK, IRE1, and GRP-78 genes, which are endoplasmic reticulum (ER) stress markers, are presented in Figure 7A–D. The obtained data showed that VCR activates ATF-6, PERK, IRE1, and GRP-78 genes in liver tissue by inducing ER stress (*p* < 0.05). On the other hand, after the animals were given RJ, an improvement in ER stress occurred, and these markers were suppressed. There was no difference in the levels of IRE1 in the low dose group compared to VCR. It was observed that the high dose group suppressed the ATF-6 (*p* < 0.05) and GRP-78 (*p* < 0.05) genes more than the low dose group.

### 3.8. Effects of RJ and VCR on Relative mRNA Transcript Levels of LC3A and LC3B Genes in Liver Tissue

**Figure 8:**
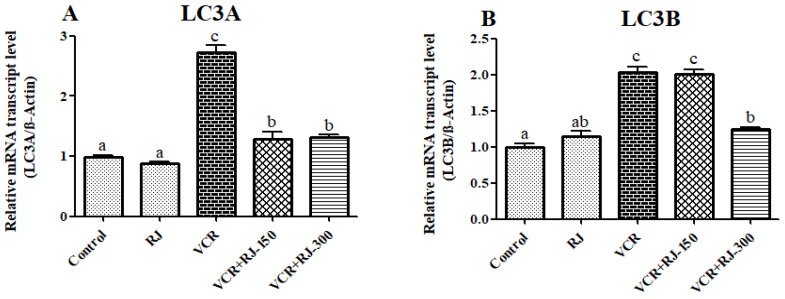
Effects of RJ and VCR treatments on LC3A and LC3B mRNA transcription levels in liver tissue. (**A**) LC3A mRNA transcript levels, and (**B**) LC3B mRNA transcript level. Values are expressed as mean ±  SD. Different letters (a–c) on the columns show a statistical difference (*p*  <  0.05).

Relative mRNA transcript levels of LC3A and LC3B genes were analyzed to determine autophagy status in liver tissue and the results are summarized in Figure 8A,B. According to the data obtained, it was observed that VCR upregulated LC3A and LC3B in liver tissue (*p* < 0.05), while RJ administration suppressed these autophagic genes. While it was determined that low dose was not effective on LC3B, it was observed that there was no difference between doses on LC3A.

### 3.9. Effects of RJ and VCR on Apoptosis in Liver Tissue

Western blot analysis was performed to assess the degree of apoptosis and inflammation in liver tissue. According to the results, the pro-apoptotic factors Bax and Caspase-3 were examined, and when compared to the Control group, the VCR group exhibited an increase of approximately 1.6-fold and 1.26-fold, respectively. In the RJ-300 group, the severity of apoptosis was reduced, with Bax and Caspase-3 protein expression levels decreasing by 39% and 36%, respectively, compared to the VCR group (*p* < 0.05).

Regarding the expression of Bcl-2, an antiapoptotic factor, the VCR group showed a 30% reduction compared to the Control group (*p* < 0.05). However, with the administration of 300 mg/kg RJ, the previously decreased Bcl-2 protein levels increased approximately 1.3-fold, indicating an attenuation of apoptosis.

**Figure 9:**
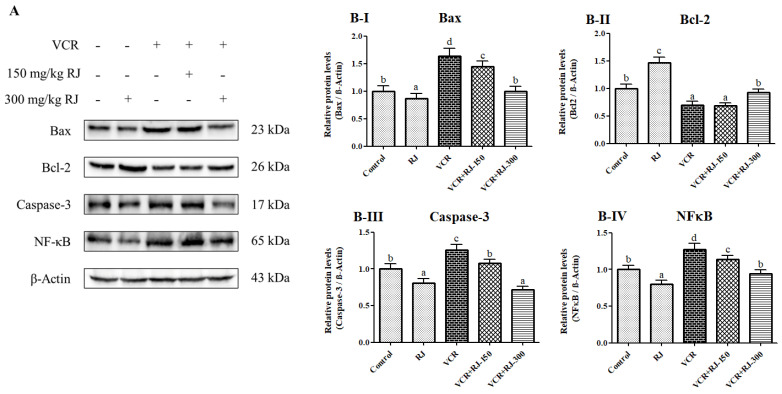
Effects of VCR and RJ on apoptosis-related protein expression levels in liver tissue. (**A**) Western blot analysis results of Bax (23 kDa), Bcl-2 (26 kDa), caspase-3 (17 kDa), and NF-κB (65 kDa) protein expression levels. β-Actin (43 kDa) protein expression level was examined as a loading control. Comparative expression levels of (**B-I**) Bax, (**B-II**) Bcl-2, (**B-III**) Caspase-3, and (**B-IV**) NF-κB protein expressions were determined using the Graphpad Prism 5 program. (Columns indicated with different letters indicate a statistical difference at the *p* < 0.05 level according to Tukey’s multiple comparison test).

Western blot analysis of NF-κB, a key marker of inflammation and a central activator of multiple pathways, revealed that the protein levels in the RJ-treated group were similar to those in the Control group. However, the protein levels in the VCR-treated group were approximately 1.3-fold higher than those in the Control group (*p* < 0.05). Additionally, a comparison between the RJ-150 and RJ-300 groups showed a 21% difference in the NF-κB protein levels following RJ administration (*p* < 0.05). (Figure 9A,B-I–B-IV).

## 4. Discussion

Vincristine is an important chemotherapeutic agent that is administered intravenously for treatment. Intravenously administered VCR is primarily metabolized by CYP3A in liver tissue. This metabolism may lead to liver damage induced by VCR [8]. Liver damage is assessed by measuring liver-specific parameters, such as AST, ALP, and ALT, which are released into the bloodstream by hepatocytes. The levels of these enzymes increase in the blood during liver injury [32]. In a study conducted by Akinrinde, et al. [33], a significant increase in serum AST, ALP, and ALT levels was observed in rats following VCR treatment. Similarly, Ghanbari, et al. [34] reported an increase in AST, ALP, and ALT levels in STZ-administered rats, while the administration of RJ to the same groups resulted in a significant reduction in AST, ALP, and ALT levels. Consistently, in our study, AST, ALP, and ALT levels increased in VCR-treated groups, whereas RJ administration led to a significant reduction in these enzyme levels.

Reactive oxygen species (ROS) are natural components involved in cellular processes, such as defense, signal transduction, gene expression, and cell growth. However, excessive ROS production can lead to oxidative stress, causing damage to lipids, proteins, and DNA [35]. Consequently, ROS play a role in the development of various diseases, including cancer, diabetes, neurodegenerative disorders, and liver diseases [36]. To prevent such detrimental effects, cells have developed an antioxidant defense mechanism to maintain intracellular ROS levels within a controlled range. Key enzymatic and nonenzymatic molecules, such as GPx, SOD, CAT, and GSH, play a crucial role in the active antioxidant defense system [37]. Although intracellular ROS levels are regulated through intrinsic processes, exposure to chemotherapeutic agents like VCR can lead to an unexpected and rapid increase in ROS production. This excessive ROS accumulation may suppress the activity of antioxidant molecules, including GPx, SOD, CAT, and GSH [38,39]. For example, in a recent study, VCR was administered to rats and a significant decrease in SOD, CAT, and GPx enzyme activities and GSH levels in liver tissue was observed compared to the control group [11]. Additionally, Gu, et al. [40] reported that RJ possesses significant antioxidant capacity. Previous studies have also reported that the antioxidant activity of RJ is not only due to its hydroxyl radical scavenging activity but also to the effect of RJ on the inhibition of enzymes that increase the peroxidation of endogenous lipids and the expression of cytochrome P450, one of the intracellular sources of oxygen radicals [41,42]. In this study, it was shown that RJ administration caused an increase in the activity/levels of antioxidant molecules, such as SOD, CAT, GPx, and GSH, and a decrease in MDA levels compared to VCR-treated groups. In the LC-MS/MS analysis of RJ, 53 types of phenolic compounds were investigated and it was determined that RJ contains 15 types of strong phenolic compounds with antioxidant properties (quinic acid, fumaric acid, gallic acid, 4-OH benzoic acid, p-coumaric acid, aconitic acid, protocatechuic acid, quercetin, caffeic acid, chlorogenic acid, chrysin, acacetin, luteolin, genistein, and apigenin). For this reason, it is thought that the rich antioxidant properties of royal jelly originate from these compounds, and there are many studies in the literature that support this [43,44,45,46]. Moreover, the rich antioxidant properties of RJ can be attributed to short-chain peptides, fatty acids (trans-10-hydroxy-2-decenoic acid), minerals (Fe, Zn, and Cu), and some vitamins (A, E, and C) in its structure [47].

Chemotherapeutic agents have an important role in stimulating and modulating the immune response [48]. Various chemotherapeutic agents, including VCR, cause inflammation in the nervous system, liver, and kidney by stimulating pro-inflammatory cytokines [49,50]. These inflammatory factors are downstream signals of a transcription factor, NF-κB. The NF-κB cleaves from IκB in the cytosol and translocates to the nucleus and regulates the expression of up to 500 genes, including pro-inflammatory cytokines, such as TNF-α and IL-1β [51,52,53]. In a previous study, it was reported that VCR contributes to neuropathic pain by upregulating TNF-α, IL-1β, IL-6, IL-18, and COX-2 genes in L4-L5 spinal cord segments; however, mitoquinone treatment can alleviate neuropathic pain by suppressing these genes [54]. In another study, it was noted that there were significant increases in NF-κB levels in liver tissue after VCR administration, but quercetin decreased NF-κB levels [11]. In the present study, it was observed that NF-κB, TNF-α, and IL-1β genes were upregulated after VCR treatment in liver tissue, but RJ suppressed these genes and protected the liver from the inflammatory effect of VCR.

The JAK/STAT signaling pathway plays a crucial role in immune regulation and tumor progression by exhibiting hyperactivity in certain cancer types [55,56]. JAKs are a family of membrane-associated proteins that become activated upon extracellular signal reception and subsequently transmit this signal intracellularly by activating STATs [57]. STATs, in turn, translocate the signal into the nucleus, enhancing specific transcriptional expressions and promoting cytokine release [32]. Consequently, the JAK/STAT pathway plays an immunoregulatory role [58]. The JAK2/STAT3 signaling pathway has been reported to play an important role in cell proliferation and apoptosis [59]. Activated STAT3 not only exhibits antioxidant properties but also stabilizes the mitochondrial membrane. Moreover, STAT3 is recognized as an antiapoptotic factor because it regulates various apoptosis-related genes, such as Bcl-2 and Bcl-xL. During cellular stress, JAK2/STAT3 can be phosphorylated, thereby inducing protective and survival signals and limiting the extent of damage [60]. A previous study has shown that the JAK2/STAT3 pathway plays a vital role in hepatic ischemia/reperfusion injury [61]. Our data revealed that in groups treated with VCR, JAK2 and STAT3 levels were significantly elevated compared to the control. However, when VCR was combined with RJ, both the VCR + RJ-150 and VCR + RJ-300 groups exhibited a significant reduction in JAK2 and STAT3 levels compared to the VCR-only group.

The majority of newly synthesized proteins acquire their three-dimensional structure in the ER lumen before they are localized to target organelles or the cell surface. Some physiological conditions or stimuli increase the demand for protein folding, and in this case, an imbalance occurs between the protein folding load and the capacity of the ER, causing the accumulation of unfolded or misfolded proteins. To overcome this situation, eukaryotic cells have developed the UPR, which alters the cell’s transcriptional and translational programs [62]. UPR signaling cascades are triggered by protein sensors in the ER of mammalian cells. These sensors are ATF-6, PERK, and IRE1. Each of these sensors has different tasks [63,64]. At rest, it is kept in an inactive state by ER chaperones, such as HSPA5 and GRP-78. When ER stress occurs, chaperones bind to unfolded or misfolded proteins, leading to the release of ER stress sensors [65]. On the other hand, if the protein folding defect in the cells is not resolved, the UPR triggers apoptosis to protect the organism [62]. Previous studies have reported that various chemotherapeutic agents, including vincristine, trigger apoptosis due to ER stress and cause dysfunction in tissues [23,66,67]. Similarly, in the presented study, it was observed that VCR triggered ER stress in liver tissue and upregulated the ATF-6, PERK, IRE1, and GRP-78 genes. On the other hand, it was determined that RJ treatment could provide protection against apoptosis by suppressing ER stress in liver tissue.

Autophagy is a cellular process responsible for the breakdown of excess or abnormal long-lived cytosolic proteins and organelles within lysosomes [68]. Autophagy plays an important role in maintaining the balance between cell survival and cell death. Its cytoprotective property has been attributed to the elimination of damaged organelles. For example, autophagy is thought to play a critical role in ER stress caused by the accumulation of misfolded proteins [69]. Increasing evidence indicates that many toxic agents, including chemotherapeutic agents, can induce autophagy in tissues, such as the liver, brain, and testis [70,71,72]. In the current study, it was concluded that the autophagic markers LC3A and LC3B [63,73] expressions were upregulated, similar to ER stress markers, after VCR application; however, these markers were suppressed after RJ treatment.

Hepatotoxicity caused by chemotherapeutic agents is closely associated with the activation of apoptotic pathways involving key proteins, such as Bax, Bcl-2, p53, and various caspases [74,75]. Bcl-2 and Bax are key regulators of apoptosis. Bcl-2 has been reported to inhibit apoptotic signaling by counteracting Bax’s promotion of cell death [76]. Chemotherapeutic agents increase mitochondrial outer membrane permeability, particularly by causing an increase in Bax/Bcl-2 ratios and cytochrome c release. It has been reported that activation of caspase-3 and caspase-9, resulting in cytochrome c release into the cytoplasm, triggers apoptosis [74,75]. In this study, the effects of RJ at doses of 150 mg/kg and 300 mg/kg on Bax, Bcl-2, and Caspase-3 protein expression in VCR-induced liver toxicity were investigated. Western blot analyses and quantitative assessments revealed that VCR administration significantly increased Bax and Caspase-3 levels while reducing Bcl-2 levels. This suggests that VCR induces apoptosis in hepatocytes. As reported in the literature, VCR is known to activate apoptotic pathways by triggering oxidative stress and inflammatory responses [11]. On the other hand, RJ administration mitigated VCR-induced apoptotic protein alterations in a dose-dependent manner. Bioactive compounds in RJ, including flavonoids, proteins, and free fatty acids, are thought to regulate the mitochondrial pathway and suppress cellular apoptosis.

## 5. Conclusions

In conclusion, our findings revealed that RJ reduces VCR-induced liver toxicity. RJ, especially due to its rich phenolic content, maybe a promising supplement to protect against organ damage due to its antioxidant, anti-inflammatory, and antiapoptotic activities. However, experimental animal studies on the protective effects of RJ against side effects caused by chemotherapeutic drugs are limited and clinical human studies are almost nonexistent. Therefore, further experiments (in vitro, animal research, and clinical trials) and validation will be required to prove the mechanism of action of RJ.

## Figures and Tables

**Table 1:** Sequences of primers.

Gene	Sequences (5′-3′)	Length (bp)	Accession No.
NF-κB	F: AGTCCCGCCCCTTCTAAAAC R: CAATGGCCTCTGTGTAGCCC	106	NM_001276711.1
IL-1β	F: ATGGCAACTGTCCCTGAACT R: AGTGACACTGCCTTCCTGAA	197	NM_031512.2
TNF-α	F: CTCGAGTGACAAGCCCGTAG R: ATCTGCTGGTACCACCAGTT	139	NM_012675.3
ATF-6	F: TCAACTCAGCACGTTCCTGA R: GACCAGTGACAGGCTTCTCT	130	NM_001107196.1
PERK	F: GATGCCGAGAATCATGGGAA R: AGATTCGAGAAGGGACTCCA	198	NM_031599.2
IRE1	F: GCAGTTCCAGTACATTGCCATTG R: CAGGTCTCTGTGAACAATGTTGA	163	NM_001191926.1
GRP78	F: CATGCAGTTGTGACTGTACCAG R: CTCTTATCCAGGCCATATGCAA	143	NM_013083.2
LC3A	F: GACCATGTTAACATGAGCGA R: CCTGTTCATAGATGTCAGCG	139	NM_199500.2
LC3B	F: GAGCTTCGAACAAAGAGTGG R: CGCTCATATTCACGTGATCA	152	NM_022867.2
β-Actin	F: CAGCCTTCCTTCTTGGGTATG R: AGCTCAGTAACAGTCCGCCT	360	NM_031144.3

**Table 2:** LC-MS/MS results of RJ.

No	Analytes	Royal Jelly (mg Analytes/g Sample)	No	Analytes	Royal Jelly (mg Analytes/g Sample)
1	Vanillin	ND	28	Ferulic acid	ND
2	Daidzin	ND	29	Salicylic acid	ND
3	Piceid	ND	30	Cyranoside	ND
4	Coumarin	ND	31	Miquelianin	ND
5	Hesperidin	ND	32	Isoquercitrin	ND
6	Quinic acid	6.573	33	Rutin	ND
7	Fumaric acid	3.927	34	Genistin	ND
8	Aconitic acid	0.043	35	*O*-Coumaric acid	ND
9	Gallic acid	0.017	36	Ellagic acid	ND
10	Protocatechuic acid	0.043	37	Rosmarinic acid	ND
11	Gentisic acid	ND	38	Fisetin	ND
12	Epigallocatechin	ND	39	Cosmosiin	ND
13	Protocatechuic aldehyde	ND	40	Quercitrin	ND
14	Catechin	ND	41	Astragalin	ND
15	Chlorogenic acid	0.019	42	Nicotiflorin	ND
16	Tannic acid	ND	43	Daidzein	ND
17	4-OH Benzoic acid	2.910	44	Genistein	0.004
18	Epigallocatechin gallate	ND	45	Quercetin	0.037
19	Cynarin	ND	46	Luteolin	0.005
20	Vanilic acid	ND	47	Hesperetin	ND
21	Epicatechin	ND	48	Naringenin	ND
22	Caffeic acid	0.023	49	Kaempferol	ND
23	Syringic acid	ND	50	Apigenin	0.003
24	Syringic aldehyde	ND	51	Amentoflavone	ND
25	Epicatechin gallate	ND	52	Acacetin	0.011
26	*p*-Coumaric acid	0.332	53	Chrysin	0.012
27	Sinapic acid	ND			

ND: Not Detected.

## Data Availability

All study results are available and raw data are available from the corresponding authors.
